# The nature of posttraumatic nightmares and school functioning in war-affected youth

**DOI:** 10.1371/journal.pone.0242414

**Published:** 2020-11-25

**Authors:** Gerlinde C. Harb, Jon-Håkon Schultz

**Affiliations:** 1 Corporal Michael J. Crescenz VA Medical Center, Philadelphia, PA, United States of America; 2 Arctic University of Norway, Tromsø, Norway; Koc University School of Medicine, TURKEY

## Abstract

Children and adolescents who have experienced traumatic events demonstrate a variety of posttraumatic symptoms, including recurrent nightmares, as well as adverse reactions in the school setting. The current study examined nightmare symptoms, posttraumatic stress, sleep disturbance, and self- and teacher-reported school functioning of 64 youths in the Gaza Strip, ages 12 to 16, who have lived through three wars and experience ongoing conflict and political insecurity. Students were treatment-seeking for sleep-problems and reported, on average, five nightmares per week for an average of three years, with concomitant disrupted sleep, fear of going to sleep, and not feeling rested in the morning. Both teachers and students reported that participants exhibited impaired academic functioning and daytime sleepiness. The content of the students’ nightmares demonstrated frightening themes of being under attack and loss of self-efficacy/control; threat levels were high, and almost 60% included the threat of death. Approximately half of the nightmares included surreal elements in addition to more realistic scenes of violence. Participants in the study demonstrated substantial posttraumatic sleep problems with intensely distressing, frequent and chronic nightmares, andnightmare symptoms were associated with impairment in school functioning. Given the disruptive and distressing nature of these students’ nightmare disturbance, we suggest that increasing self-efficacy in relation to the experience of recurrent nightmares may be a good point of intervention with these recurrently traumatized youth. Thus, increasing the understanding of students’ nightmare symptoms may lead to ameliorating the suffering of youths in war zones and may have positive effects on their school functioning.

## Introduction

Children and adolescents living in war-torn environments frequently experience stressful conditions and are exposed to multiple traumatic events, which can severely challenge their mental health and their emotional and even physical development (e.g., [1]). These posttraumatic symptoms in turn affect children’s capacity to learn in school. A systematic review of 83 studies showed that children with exposure to recurrent and/or severe traumatic events are at significant risk not only of cognitive impairment and social-emotional-behavioral problems, but also academic difficulties, compounding the impact on their well-being. [2]. The fifth edition of the Diagnostic and Statistical Manual of Mental Disorders [3] cites behavioral problems, impaired cognitive functioning, somatic distress, and psychological reactions, including Posttraumatic Stress Disorder (PTSD), as sequelae of traumatic exposure.

The ongoing military siege of the Gaza Strip has been enforced since 2007, with tight controls of the movement of goods and people causing deprivation of basic needs among almost two million Palestinians [4]. Since the start of the siege, the civilians of Gaza have been exposed to three major wars: in 2008–2009, 2012, and 2014. In addition, between the end of the Israeli military operations in January 2009 and November 2012, the United Nations Office for the Coordination of Humanitarian Affairs (OCHA) recorded an average of ten “limited escalations” per year in the Gaza Strip, each lasting an average of two and a half days. As an example of the population’s potential exposure to traumatic events, the 2014 war lasted 54 days and carried a reported death toll of 2,251, including 299 women and 551 children; 11,231 individuals were wounded, including 3,540 women and 3,436 children; 18,000 housing units were destroyed; and about 500,000 people were displaced [5]. Studies have documented that Gazan children have experienced displacement and/or witnessed death, shelling, and combat situations [6]. Studies have further indicated that 64 to 67% of children in Gaza demonstrate clinical levels of posttraumatic stress symptoms [7, 8].

Following the 2014 war, teachers and administrators in schools in the Gaza Strip voiced concerns to the United Nations Relief and Works Agency for Palestine Refugees in the Near East (UNRWA) that a large number of students were exhibiting adverse reactions in the school setting, such as falling asleep during class, difficulty concentrating, and poor academic performance. The fundamental role of the sleep disturbance in adult PTSD is well-documented [9, 10]. Similarly, a recent review of the more limited literature on sleep disturbance in pediatric PTSD [11] concluded that there is strong evidence for higher rates of sleep problems, including nightmares (i.e., frightening dreams that awaken the dreamer), in trauma-exposed children and adolescents, including those with PTSD. Generally, nightmares are more common in children than in adults, with adolescents experiencing fewer nightmares than younger children [11]. Similarly, posttraumatic nightmares appear to be more frequent, almost universal, after trauma exposure in children and youth compared to adults [12]. Furthermore, Wittman and colleagues (2010) underscored the importance of posttraumatic nightmares in children by demonstrating, in traffic-accident survivors, that posttraumatic nightmares, in particular replicative nightmares (those replaying the original traumatic event), were strongly predictive of the later development of PTSD [12].

It has been suggested that a focus on the treatment of posttraumatic sleep problems in adults may provide an easier entry into trauma treatment, with diminished perception by the patient of stigmatization [13]. Few studies have focused on treating posttraumatic sleep problems directly in children or adolescents [14, 15] despite the recognition of the importance of sleep and its role in development, including the development of chronic posttraumatic symptoms [16]. This is an area of great importance, because interventions directly addressing sleep and nightmares in traumatized children have great potential as a first-line treatment: First, sleep- and nightmare-focused interventions allow for easy identification of a clinical population in schools due to easily assessed and circumscribed symptoms. Second, such treatments are associated with less cultural stigma for students and their families, which is otherwise common in relation to mental health treatment [e.g., 17]. And third, they directly address a core problem in posttraumatic adjustment that directly interferes with youth’s functioning.

In response to the needs of youths in Gaza, the Norwegian Refugee Council (NRC) together with UNRWA developed and began to implement a trauma-focused, school-based intervention, the Better Learning Program (BLP) [18], in more than 200 UNRWA primary and middle schools in the Gaza Strip. BLP, which specifically targeted students with recurrent posttraumatic nightmares, has been in operation since 2012 and provided participants for the current phenomenological study.

The goal of the current study was to examine the nature of posttraumatic nightmares in young adolescents in primary school in Gaza. The extant literature on nightmare content in traumatized children (reviewed in [12]) is inconsistent, with some studies suggesting increased symbolism and others increased realistic depictions and fewer bizarre elements. Furthermore, prior research on the dreams of Palestinian youths focused on specific theories of dreaming [19] or examined dreams of the general population of Gazan children [20]. The current description of the phenomenology of posttraumatic nightmare symptoms in youths with reported sleep disturbance and functional impairment in school is an initial step in ameliorating the problems experienced by many children in conflict zones. Furthermore, accurately describing the problem is essential for maximizing the efficacy of interventions. This paper aims to (1.) describe the characteristics of students’ nightmares (content, emotions, and themes) and (2.) explore the relationship between nightmare symptoms, other symptoms of PTSD, and student- and teacher-rated school functioning.

## Method

### Participants

Participants for the current study were 64 students (63% female) ranging from 12 to 16 years of age who were enrolled in BLP in February 2016 at eight schools in the Gaza Strip, which were chosen by UNRWA for implementation of the BLP program during that time. The study's protocols were reviewed by the heads of the Department of Mental Health and the Department of Education in Gaza who recommended the research project for final ethical approval by the Board of Directors. Further, the director of the UN Gaza Field office approved the research project to be carried out in selected UN schools in Gaza. Participation in the research project was voluntary, and enrollment required written parental consent. Participants provided their informed consent for study participation after procedures were fully explained to them. Inclusion criteria (as assessed in the baseline screening) for the BLP were self-reported posttraumatic nightmares, two or more nights per week, over three months or more, interfering with the student’s daily functioning (criteria used in other trials of treatments of posttraumatic nightmares, e.g. [21]). All participants had lived through the three wars during their school years and had heard the intense noise of war and seen graphic television pictures of war scenes, including dead people, violence, bombing, and destroyed buildings. About half of the students had directly witnessed such scenes, and many had evacuated their homes and lived in shelters during the most recent war. Many reported death or injuries among family, extended family, and friends.

A bachelor’s-level counselor at each school had extensive training in the recruitment and screening procedures and led the screening interviews. The counselors gave briefings in classrooms, including psychoeducation about posttraumatic nightmare experiences, and invited students with recurrent trauma-related nightmares to sign up for the BLP intervention to reduce their nightmares. Interested students were individually screened using a structured interview assessing nightmare frequency, intensity, and content, as well as school functioning and general well-being. Participation in the assessment and group intervention was voluntary, and enrollment required parental consent. The current study reports on nightmare phenomenology assessed before (self- and teacher report) and during the first individual session of the BLP program (nightmare script write-out), not on BLP treatment outcome (manuscript in preparation).

### Measures

#### Self-report of PTSD, sleep/nightmares, and school/social functioning

The children were administered a self-report questionnaire assessing their sleep and nightmares, including hours spent in bed, sleep latency, waking after sleep onset, feeling rested upon arising (using a 5-point scale from 1 = *never* to 5 = *almost every night*), and frequency and duration of nightmares. The questionnaire also assessed students’ fear of sleep, the nature of their nightmares, and whether the nightmare problem had been previously discussed with others. Furthermore, students were asked to rate their school and social functioning on a 10-point scale (0 = *not at all* to 10 = *all the time*), with questions about concentration, self-assessment of school performance, and feelings of happiness and social connectedness. Five self-rated school functioning items were summed to create a reliable total *Student Functioning* score (coefficient alpha = .79). Nightmare intensity was measured with the 3-item *Nightmare Intensity Scale* developed by Harb and colleagues (manuscript in preparation) assessing intensity of imagery, sensory details, and emotional experience in nightmares.

The *Children’s Impact of Events Scale—13 (CRIES-13*; [22]) was used to assess posttraumatic symptoms. The CRIES-13 is 13-item self-report measure consisting of four items measuring intrusion, four items measuring avoidance, and five items measuring arousal characteristic of PTSD as defined by the *DSM-IV* ([23]; the current diagnostic manual at the time of the inception of the study). The items are rated on a four-point scale (0 = *not at all*; 1 = *rarely*; 3 = *sometimes*; 5 = *often*) and summed to form three subscales. Psychometric data suggest satisfactory internal consistency (Cronbach alphas for Intrusion = 0.70; Avoidance = 0.73; Arousal = 0.60; and Total = 0.80). A combined score (Intrusion + Avoidance subscales) of 17 or more has been found to correctly classify PTSD in 90% of child survivors of a disaster [24]. Due to an administrative error, only 40 of the 64 students, and only girls, were asked to complete the CRIES-13.

#### Teacher report of school/social functioning

Teachers completed a brief questionnaire about their students. They were asked to rate the following on a 10-point scale: the student’s happiness and social connectedness, ability to perform consistently with her/his scholastic capabilities, level of concentration, behavioral problems, and demonstrated sleepiness (and actual falling asleep in class). Six teacher-rated school functioning items were summed to create a reliable total *Teacher Functioning* score (coefficient alpha = .83). Items assessing sleepiness were excluded from this total score to avoid biasing the analyses with other sleep-related measures. All questionnaires were translated from English into Arabic and back translated by certified translators.

### Nightmare scripts and nightmare coding

During the individual part of the BLP treatment, each student was asked to provide a written script of a recurrent nightmare. The majority wrote the script themselves; in six cases the student dictated the script to a counselor. The counselors supported and encouraged the students and provided the following instruction (translated to Arabic by certified translators):

*Please write down your nightmare story*. *Write the nightmare in all its detail (including what you are seeing, feeling, hearing, smelling in the nightmare)*. *Please think about how it usually starts and write the nightmare down as you usually dream it, to the point when you usually wake up*. *If your nightmare is based on something that has happened to you, make sure you write the nightmare story you dream, not the story of the event as it actually happened*.

All nightmare scripts were translated into English by two certified translators. Of the 64 students, 61 provided nightmare scripts; the three others were missing due to administrative errors, and three additional nightmare scripts were not rated due to lack of sufficient detail/length of the script. Fifty-eight scripts (91%) were coded and analyzed (included students did not differ from excluded students in any significant way).

Nightmares and dreams were coded using an iterative process. Two doctoral-level psychologists (GH and JHS) used the coding instrument developed by Harb and colleagues (2012) to rate nightmare content [25]. Items assessed setting, scenes of life threat/injury to self and others, general scenes of injury/death, and sensory (auditory, olfactory, tactile/sensation, and visual) details, as well as applicable nightmare content themes (described in tables below, slightly modified from the adult-veteran version of the coding instrument). Independent ratings were made on the presence or absence of specific nightmare characteristics, and consensus ratings were used for analyses in cases of disagreement. Determination of primary or secondary dream themes were made according to affective salience and the relative amount of text focused on the theme. One primary theme was coded for each dream, and multiple secondary themes were recorded as applicable.

### Analyses

Descriptive analyses of clinical and demographic characteristics were completed. The primary analyses focused on the links between nightmare and dream characteristics and pre-treatment sleep and nightmares and other clinical characteristics. Further analysis examined the relationships among the following variables: self-reported nightmare frequency and intensity, self-reported school/social-emotional functioning and teacher-reported school/social functioning as well as PTSD symptomatology. Due to the smaller number of participants who completed the PTSD measure, those analyses were conducted on the participants (n = 40) who had data on all measures. Significance of relationships was explored using Pearson correlations with Bonferroni corrections. We hypothesized that nightmare frequency and intensity would be related to functional outcomes, both self- and teacher-reported. In addition, we hypothesized that the exploratory subsample analysis will show a relationship between overall PTSD symptomatology and school functioning.

## Results

### Demographic and clinical characteristics

The 64 participants included in the current study ranged in age from 12 to 16 with an average age of 13.5 (*SD* = 0.94) and included 40 females (63%) and 24 males (37%). The students exhibited disturbed sleep, as indicated by an average 50 minutes of nightly sleep latency (*SD* = 37.9; mode = 60 minutes), waking after sleep onset several times a week to almost every night (average 4.4/5; *SD* = .59) and feeling rested in the mornings on average only once per week (3.12 on 5-point scale from 1 = *never* to 5 = *almost every night*; *SD* = 1.32).

Students reported an average of 4.7 nights with nightmares per week (*SD* = 1.36, mode = 6) and an average duration of 3.1 years with a nightmare disturbance (*SD* = 1.5; mode = 3, range 1–7 years) during which they experienced less than 2 weeks without nightmares. Furthermore, 81.3% of students reported fear of going to sleep because of nightmares, and only 20.3% indicated an ability to return to sleep after nightmares. Of these students with nightmares, 61.3% had previously told someone about their nightmare problems, but only 6.3% had told a teacher.

Of the 40 students (girls) who completed the CRIES-13, 90% (*n* = 36) had scores in the PTSD range (Mean *Intrusion+Avoidance* score = 27.2, *SD* = 9.02). The average CRIES-13 Total score was 43.3 (SD = 15.23), ranging from 8 to 65. The 10% of students who did not score in the PTSD range exhibited nightmares at a similar frequency (*F* = 1.39, *p* = .39) and intensity as the PTSD participants (*F* = 0.01, *p* = .75).

In terms of functional impairment, students reported on a 10-point scale (0 = *not at all* to 10 = *all the time*) that they often felt they could not do their best in school and had difficulty concentrating (average 5.9 [*SD* = 2.17] and 4.9 [*SD* = 2.42], respectively). They also reported not feeling very happy (4.5, *SD* = 2.1), although they did like being at school (7.2, *SD* = 2.55). Using the same scale, teachers rated students’ functioning similarly in terms of their ability to do their best and to concentrate (5.2 [*SD* = 2.25] and 4.5 [*SD* = 2.13], respectively). Teachers also expressed concern about students and the behavior problems they exhibited in the classroom. Finally, teachers noticed that approximately half (49%) of the students appeared sleepy in class at least once a week, with 27% of students appearing sleepy several times per week. Furthermore, they reported that 19% of students actually fell asleep in class at least once a week.

### Nightmare characteristics: Content, emotions and themes

In terms of nightmare characteristics, 79.7% of the youths reported recurrent similar nightmares. Nightmares replaying actual events or nightmares of their worst life experiences were common (68.8% and 60.9%, respectively). In terms of emotional content, the most frequent primary emotion experienced in the students’ nightmares (per self-report) was fear: 77% reported predominant feelings of anxiety, fear, or horror. The second most commonly reported primary emotion was grief (20%), followed by helplessness (19%). Other salient emotions included despair, anger, guilt, and sadness (12, 5, 3 and 3% respectively).

Table 1 shows details of the characteristics of the students’ nightmare scripts. Approximately half (48%) of the nightmares contained surreal or unreal dream elements, such as skeletons, monsters, ghosts or talking animals. Animals were present (26%) mostly as scary and aggressive dogs, snakes, cats or lions. Threatening people appeared as shadows, faceless or with deformed faces, with masks and strange voices. With regard to sensory details included in these nightmare scripts, the students’ nightmares most often included auditory (55%) or color (40%) detail.

**Table 1:** Nightmare characteristics among war-affected youth.

	% of *N* = 58
Setting	
• Violence setting	47
• War/combat setting	16
Scenes of death	45
• Death/serious injury of family member	16
• Death/serious injury of other people	24
Life threat to the dreamer	90
• Dreamer injured	31
• Dreamer dies	10
Dreamer kills someone/something	5
Surreal/unreal dream elements	48
• Animals	26
Sensory detail	
• Sound	55
• Color	40
• Sensations	31
• Smell	16

Most nightmares featured the dreamer’s life under threat (90%), many were set in the midst of violence or war (46.6% and 15.5%, respectively) and/or included gruesome scenes of death (45%) with serious injuries to or death of family members (16%), other people (24%), or the dreamer him- or herself (41%). Depiction of perpetration of violence by the dreamer him- or herself was rare (5%). The average degree of threat was 4.1 (*SD* = .85) on a five-point scale. Specifically, 69% dreamed of immediate or catastrophic threat to their lives (4 or 5). Table 2 shows examples of realistic versus symbolic dreams, both containing high degrees of threat to the dreamer.

**Table 2:** Examples of war-affected youth’s nightmares: Realistic and symbolic nightmares.

Realistic	*I was walking in the street during the horrible war days*. *I was afraid and running away*. *On this day*, *my family wanted me to flee for fear of my life*. *With every step I was taking*, *a missile fell down bombing people and shattering them into pieces*. *I was very afraid*.
Symbolic	*All people*, *even my family members*, *were ghosts*, *but I managed to identify some faces*. *They were chasing me*. *They were colored in red*, *blue*, *green*, *yellow*, *and all colors*. *Whenever they try to touch me*, *I run so fast because if they touch me*, *I will become just like them*. *I don’t want to be like them*. *They had no feet and they were floating in the air*. *When I see them*, *I feel terrified because they are scary and they are too loud and annoying*. *My father*, *mother*, *siblings*, *and all my family were like them*. *I was alone*. *I was the only human*. *I was really scared*. *When I wake up*, *I find myself sweating a lot with racing heartbeats*.

More than half of the dreamers (54%) were chased by someone or something. In a typical chase dream, the dreamer is either not able to escape because they cannot move fast enough, or they manage to escape only to face a new potentially deadly threat, as exemplified in the following excerpt.

*“I am looking around, there is no one around me. Then I see the shape of a person, but I can’t really see him, only the shadows*. *I don’t know him*. *He starts chasing me, so I run very fast and my heart beats are so hard. I feel scared and my body trembles. I feel pain in my legs. I keep running until I get up on the roof of my family’s house*. *I look down from the roof and I see the man. I feel so scared and then I fall off the roof*.*”*

Table 3 exhibits the frequency of thematic nightmare contents, specifically mutually exclusive primary themes and ancillary secondary themes. The most common primary themes for nightmares were being under attack (22%) and experiencing a personal sense of lack of self-efficacy (21%), followed by a feeling of lack of control over external events (16%) and being chased (14%). In addition to primary themes, the most commonly expressed secondary themes were lack of control (62%), fear of death (47%), lack of self-efficacy (40%), and being chased (40%). Rated as either primary or secondary, 59 to 78% of nightmares demonstrated themes of lack of control, lack of self-efficacy, and fear of death.

**Table 3:** Primary and secondary nightmare themes (*n* = 58).

	% primary	% secondary	% primary or secondary
Under attack	22	26	48
Lack of self-efficacy	21	40	61
Lack of control	16	62	78
Being chased	14	40	54
Fear of death	12	47	59
Danger	12	17	29
Loss of family member/close friend	3	10	13
Aloneness	0	16	16
War is gruesome	0	5	5
Sadness	0	3	3
Why is this happening?	0	3	3

*Note*: One primary theme and an average of three secondary themes were rated for each nightmare.

With lack of control and self-efficacy as predominant primary themes, there were only a few instances when the dreamer reported receiving help or attempts of help from others. In one such example, the dreamer was chased and almost killed by several people hitting her hard with long sticks. Her mother appeared and managed to chase away the attackers. The girl was left bleeding and helpless in the street until an ambulance arrived to rescue her: “Suddenly, the ambulance came and there was a big dog in it. The dog started biting me.” There was nothing the dreamer could do to influence her own situation, and help from others (i.e. her mother and the ambulance) was also not able to eliminate the threat in the nightmare.

The nightmare below is an example of a primary theme of lack of control over external events due to the various and continuous threats that keep appearing. Secondary themes were identified as being under attack, being chased, danger and loss of family members.

*“It was war and my family was running away from home*. *While they were in the street, a rocket went into my brother's head and exploded, and all of them died*. *I was with my youngest sister at home. A person called us, and was making fun of us and said that all my family died*. *We were crying and went to the balcony and my sister fell down and there was blood, and suddenly, the street was full of blood like a waterfall. There were arms and legs of people spread everywhere*. *Then, the sea flooded over us and the water colored with blood, and then the color become black. An old man was talking to me while I was screaming. Then I died–and my home is suddenly full of blood. The ground and the sky crackled*.*”*

Table 4 shows the outcomes of the correlational analyses examining relationships between nightmare symptomatology and school functioning. Nightmare frequency was significantly associated with reduced functioning (teacher-rated *r* = -.28, *p* = .01 and student-rated *r* = -.30, *p* = .007), and nightmare intensity with reduced student-rated functioning (*r* = -.34, *p* = .003). In an explorational analysis of the female-only subsample (N = 40), the severity of PTSD symptomatology was not related to either teacher-rated or student-rated functioning (*r* = -.06, *p* = .69; *r =* -.09, *p* = .56).

**Table 4:** Correlations of nightmare symptoms with functional outcomes.

	Nightmare frequency	Nightmare Intensity (Total Score)
Teacher-rated functioning	-.28*	-.09
Student-rated functioning	-.30**	-.34**

## Discussion

The current investigation found a significant nightmare problem in a group of treatment-seeking Palestinian youths studied in the Gaza Strip. The students, who were all willing to disclose sleep problems in a school setting demonstrated impairment in school functioning and reported having distressing nightmares on average five nights per week, significant problems going to sleep due to fear of nightmares, and difficulty returning to sleep after waking from nightmares. On average, these students felt rested only one morning per week, and it is not surprising that both teachers and students reported impairment in students’ ability to perform to their capacity, concentration, and general happiness. Furthermore, the duration of these adolescents’ nightmare disturbance (an average of over three years) suggests a chronic, unremitting nightmare problem in this group of youths. Experiencing ongoing conflict, the students were subject to frequent re-traumatization, and the insecure political situation likely prevented natural recovery from traumatic events. The dreams described in this report were clear manifestations of posttraumatic symptomatology. Nightmares have been conceptualized as indicators of incomplete processing of traumatic material and therefore high levels of posttraumatic symptoms in addition to nightmares are not surprising [26].

Fear (anxiety/horror) was by far the most frequently reported emotion in the students’ nightmares, both by general self-report and in coding of specific nightmare scripts. These results are consistent with a prior investigation of dreaming in Palestinian children, which asked self-selected volunteers to complete 14 days of dream diaries and demonstrated that, even in a less distressed sample, fear was the most common emotion reported in dreams generally [20]. Our sample, in contrast, was treatment-seeking for sleep disturbance and identified as in distress and with poor school functioning; their dream reports were of nightmares that often replayed elements of actual traumatic events. Interestingly, approximately half of these nightmares contained surreal, bizarre dream elements, different from the less surreal nightmare content of a group of adults with posttraumatic nightmares (specifically, US combat veterans [25]). Other differences included the addition of a new theme for this population (*Feeling alone/Aloneness)* and the lack of depictions of *Guilt* and *Self-blame* themes. Given that guilt themes have been found to result in reduced treatment response in veterans [13], the lack of guilt and self-blame in these adolescents’ nightmares may bode well for treating their nightmares and sleep disturbance.

The negative affective content of the students’ distressing nightmares is consistent with a recent study of the structure of dreaming in war-exposed Palestinian children, which found an association between “unpleasant, fragmented and lonely” dreams and reduced psychological well-being, and a relation between pleasant, active, and symbolic dreams and fewer PTSD symptoms [20]. Our current findings of predominant themes of lack of control or lack of self-efficacy additionally suggest that youth are experiencing a sense of being unable to act and effect change within their nightmare stories.

The nightmares exhibited very high levels of threat, with 90% of students experiencing a fear for their own life. This finding supports notions of the Threat Simulation Theory studied in this population by Valli and colleagues [19]. The authors found differences in dream content between Palestinian children living in quiet versus violent locations and concluded that traumatic experiences increase the frequency and intensity of threatening dream content. Additionally, the threatening and violent content of Gazan children’s nightmares supports the *continuity hypothesis*, which posits that the dreamer’s experiences and concerns in waking life are the main sources of dream content [27].

Contrary to our hypothesis, exploratory analyses in the sub-sample of girls who completed all measures showed that PTSD symptomatology was not significantly related to either teachers’ or students’ assessment of their school functioning. Nightmares, on the other hand, were (as expected) related to these objective and subjective assessments, as demonstrated in small to medium correlations. The teachers’ reports of behavioral and academic problems in school were related to students’ self-reported nightmare frequency: more nightmares were associated with more negatively impacted school functioning. In addition to nightmare frequency, nightmare intensity also negatively affected students’ self-assessed functioning (a finding potentially inflated due to the self-reported nature of those variables). Nightmare symptomatology appears to not only be a distressing symptom in itself but to also affect students’ daytime functioning above the effects of other PTSD symptomatology.

It has been shown that recurrent nightmares significantly impair sleep quality and daytime functioning, cause sleep avoidance, daytime sleepiness and fatigue, and affect comorbid psychiatric symptoms [28]. Youth are particularly vulnerable to posttraumatic nightmares due to their development [11]. Considering the findings in this study where adolescents reported an average of 4.7 traumatic nightmares per week with an average duration of over three years, these nightmares are likely to have a developmental impact. Further, the highly distressing imagery of these nightmares raises the importance of screening and treating students living in ongoing conflict. We suggest that increasing self-efficacy in relation to the experience of recurrent nightmares may be a good point of intervention with these recurrently traumatized youth. In adults with posttraumatic nightmares, the success of Imagery Rehearsal, a cognitive-behavioral treatment that involves rescripting problematic nightmare imagery during waking, has been attributed in part to mastering or regaining control over experiences outside the individual’s sphere of control, i.e., in their dreams [29, 30].

This study provides new insight into the characteristics of posttraumatic nightmares in youths in a high conflict area who have lived through several war situations. The selection of participants and the execution of the study were ecologically valid as the study was conducted in schools with extant staff and in the context of the school environment (i.e., there were no extra requirements of students outside of school, such as parental involvement or completion of sleep diaries). However, the study had some limitations. First, the results regarding PTSD symptomatology were limited due to an administrative error that allowed only the assessment of a sub-sample of girls. As such, this part of the project may not generalize to boys, and the smaller sample size may have led to decreased power to detect significant results. Second, purposive sampling of participants and selected schools may have limited the generalizability. Specifically, this study examined a self-selected group of students who were willing to disclose sleep difficulty in a school setting. In the absence of random sampling and/or a control group who did not disclose sleep problems, the results cannot be generalized to all youth in this setting. Third, the constraints on the length of assessments and the nature of the school-based assessment process limited the scope of the assessments available in this research project. Some variables could have been assessed with more exhaustive, previously validated measures and using multiple reporting methods, such as parent reporting and/or sleep diaries. For example, trauma exposure was not formally assessed; however, the counselors obtained the necessary information on exposure both during the screening interview and during treatment, when pupils were constructing a timeline including “scary events” that they had experienced over the course of their lives. Finally, although this was the purpose of the study, we examined the nightmares of only treatment-seeking students, thereby potentially biasing the sample towards a more distressed and symptomatic group.

The current study has revealed that posttraumatic nightmares have highly distressing imagery and consequences in traumatized youths. These nightmares are associated with significant suffering, including reduced sleep and impaired functioning at school. The study also demonstrated help-seeking behavior when students were invited to sign up for a school-based treatment targeting nightmares. There may be a willingness to address the circumscribed problem of nighttime symptoms in a culture more reluctant to discuss emotional symptomatology with professionals. Considering the limited literature on posttraumatic nightmares in children and youths, more research is needed on prevalence and treatment. In particular, war-affected students are a vulnerable group and would benefit from investigations informing the most efficacious and most accessible treatment options to help their recovery from posttraumatic nightmares and sleep problems in the context of ongoing conflict.
